# Assessment of improvement in functional outcomes between a novel knee replacement design and conventional designs in 240 patients: a randomized controlled trial

**DOI:** 10.2340/17453674.2024.42708

**Published:** 2025-01-24

**Authors:** Tero IRMOLA, Aleksi REITO, Jarmo KANGAS, Antti ESKELINEN, Mika NIEMELÄINEN, Ville M MATTILA, Teemu MOILANEN

**Affiliations:** 1 Coxa Hospital for Joint Replacement, Tampere; 2 Department of Orthopaedics and Trauma, Tampere University Hospital, Tampere, Finland

## Abstract

**Background and purpose:**

The introduction and development of new total knee arthroplasty (TKA) implant designs are industry driven. To date, an adequately powered randomized controlled trial (RCT) to provide evidence of the superiority of novel implant designs over conventional ones is often lacking. The aim of our RCT was to investigate the functional outcomes of a novel TKA implant design compared with 2 conventional TKA designs. Primary outcome was difference in the change in Oxford Knee Score (OKS) at 2 years. Secondary outcomes were Forgotten Joint Score, 15D quality of life questionnaire, UCLA activity score, and complications.

**Methods:**

We compared functional outcomes between a novel TKA implant design (Persona CR) and 2 conventional designs (NexGen CR, PFC CR). 240 patients with severe knee osteoarthritis were recruited to a pragmatic, single-center, prospective, parallel-group RCT between September 2015 and August 2018. The duration of follow-up was 2 years.

**Results:**

Of 240 randomized patients, 225 were included in the intention-to-treat analysis (mean age 61.8 years; 67.5% females). The OKS exceeded minimal clinical important difference (MCID) from baseline to 2 years in all 3 treatment groups (Persona CR: 18.9 points, PFC CR: 20.3 points, NexGen CR: 19.4 points). At 2 years the difference between Persona CR and PFC CR in the change score was –1.0 (95% confidence interval [CI] –3.6 to 1.7). Similarly, the difference between Persona CR and NexGen CR was –0.9 (CI –3.6 to 1.9). At the time of final follow-up evaluation, OKS was equivalent between groups, as CI excluded between-group differences larger than 4 points.

**Conclusion:**

We showed no clinically relevant differences in functional outcomes measured with OKS, 15D, or FJS between the 2 conventional implant designs and the novel implant design at 2-year follow-up.

During the past decade, several different TKA implant designs have been developed and launched to the market. When compared with the previous generation of modern TKAs, these new designs include more anatomical shape, finer sizing increments, and a continuum of bearing constraints. The proposed advantages of these new designs include improved patient-reported results and increased patient satisfaction. These new implant systems are often based on the legacy of their predecessors, which have been among the most implanted and best-documented designs globally. In addition to standardized off-the-shelf (OTS) implants, customized TKA implants have also been introduced [1-3].

The longevity of contemporary TKA designs is excellent with a survival rate that exceeds 95% during the first postoperative decade [4-6]. Ideally, modern implants should be introduced to the market following randomized controlled trials (RCT) and clearly show improved outcomes that justify a new implant and the increased cost of the implant compared with the costs of earlier designs.

The aim of our RCT was to investigate the functional outcomes of a novel TKA implant design (Persona CR, Zimmer, Warsaw, IN, USA) compared with 2 conventional TKA designs (PFC CR, DePuy, Warsaw, IN, USA and NexGen CR, Zimmer, Warsaw, IN, USA). The primary outcome was the change in Oxford Knee Score (OKS) at 24 months. Secondary outcomes were change in Forgotten Joint Score (FJS), pain (visual analogue scale, VAS), patient activity (UCLA), quality of life (15 D), and complications measured at 3, 12, and 24 months.

## Methods

### Study design

We conducted a parallel group randomized superiority trial with a 1:1:1 allocation ratio. Patients were randomized in 3 groups: the intervention group with a novel TKA implant design and 2 control groups, each with a conventional TKA implant design. The study was designed and conducted in accordance with the SPIRIT and CONSORT guidelines. The study protocol has been published elsewhere [7].

### Participants

All patients living in our hospital district who presented at Coxa Hospital for Joint Replacement (September 2015 to August 2018) with primary, Kellgren–Lawrence grade 3–4 knee osteoarthritis (OA) and who had decided to be operated on were assessed for eligibility by participating orthopedic surgeons alongside their routine outpatient work. Patients who met 1 or more of the following criteria were excluded from participation in the trial: unwilling to provide informed consent, > 15° varus or valgus, or > 15° fixed flexion deformity, predominantly patellofemoral OA, physical, emotional, or neurologic conditions that would compromise rehabilitation and follow-up (i.e., drug or alcohol misuse, serious mental illness, and general neurological conditions, such as Parkinson’s disease, or MS). If a patient was eligible and willing to participate, written informed consent was obtained.

Preoperative medical history was carefully documented according to routine screening prior to TKA. Preoperative planning included plain radiographs of the affected knee and standing long axis radiographs of the limb. Any history of chronic pain, longstanding pain medication, fibromyalgia, depression, anxiety, or other mental disturbances were recorded. In addition to our normal preoperative protocol (including the OKS accompanied by anchor questions), the patients were also asked to complete the 15D quality of life questionnaire, the UCLA activity score, the FJS, the pain catastrophizing scale (PCS), and brief pain inventory (BPI) questionnaires.

### Interventions

All participating surgeons were specialized in knee joint replacement. 6 surgeons performed all the surgeries using the 3 implant options. 2 conventional implant brands have been in use in our hospital since 2002 and operation with the novel implant was started at the beginning of this study. The Persona implant has an asymmetric tibial component that can be considered more anatomical than its predecessor the NexGen TKA. Patients were operated on with the implant allocated in the randomization. In this study, CR and more congruent curved (n = 37) plus and ultracongruent (UC, n = 29) inserts and 1 PS insert were used in implants when the surgeon intraoperatively preferred its use (see Supplementary data). Perioperative treatment was performed according to the routine protocol of the hospital, which includes the medial parapatellar approach. The mechanical alignment [8] technique was used, and all surgeons used the same surgical technique. The measured resection [9] technique, a combination of bony landmarks, was used to determine the proper rotation of the femoral component. When necessary, soft tissue release and ligament balancing was performed to balance gap differences and/or to achieve varus/valgus ligamentous balance. All the implanted components were cemented, and the patella was only resurfaced if there was a problem with patellar tracking. The TKAs were performed under spinal anesthesia in combination with intravenous sedation. Immediate full weightbearing was allowed, and all patients were mobilized on the day of surgery. Patients received antithrombotic prophylaxis with low molecular-weight heparin, enoxaparin, for 3 to 4 weeks postoperatively. All details of perioperative care and possible complications and reoperations were recorded using the hospital’s electronic database in a routine manner.

### Outcomes and follow-up

The primary outcome was the change in the Finnish-language version of the OKS [10] measured at 2 years. Secondary outcomes included 15D quality of life questionnaire, UCLA activity score, FJS, pain, and complications. The OKS comprises 12 items regarding pain and activities of daily living (ADL) [11-14]. The total score ranges from 0 to 48, with 48 being the best possible score. The MCID for the OKS is 5 points when comparing 2 different patient cohorts, 7–8 points when comparing the change in 1 patient, and 9 points when comparing the change in the same-patient cohort [11,15]. The 15D is a generic, comprehensive, 15-dimensional, self-administered instrument for measuring health-related quality of life [16]. The 15D questionnaire comprises 15 dimensions with 5 ordinal levels on each dimension. A set of population-based preference and utility weights is used to generate the 15D score on a 0 (being dead) to 1 (full health) scale. The generic MCID for the change of 15D scores is 0.15 [17]. The UCLA is a single-item, 10-level-scale, ranging from level 10, representing a highly physically active patient, to level 1, a patient who is dependent on others and unable to leave home [18]. The FJS consists of 12 questions and is scored on a 0–100 scale. The higher the score, the less the patient is aware of their affected joint when performing daily activities [19]. The MCID for the FJS is 10.8 points [20]. Patients were evaluated by a research physiotherapist in an outpatient clinic at 3 months, 12 months, and 24 months after surgery. Reoperation was defined as any additional surgical procedure, including revision arthroplasty, wound operations without or with arthrotomy, debridement and irrigation, and manipulation under anesthesia (MUA). Revision arthroplasty was defined as revision of any or all TKA components (femur, tibia, both, or adding patella) or revision of polyethylene insert for any reason, including prosthetic joint infection (PJI).

### Sample size

The power analyses were calculated using both the OKS (primary outcome) and the FJS (secondary outcome). With the OKS, assuming an MCID of 5 points and a conservative standard deviation (SD) of 8.8 points for 2 group comparison, the estimated sample size was 48 patients per arm (alpha = 0.05, power = 0.8). With the FJS, assuming a difference of 13 points and an SD of 25 points, the estimated sample size was 64 patients per arm (alpha = 0.05, power = 0.8). Allowing for a 10% dropout rate and a 10% addition due to skewness in the variable distribution, the required arm size was 80. Therefore, with 3 comparison arms, the total number of patients recruited into the study was 240.

### Randomization and blinding

We created a list of 240 randomization numbers to guarantee equal group sizes. Computer-generated block randomization with varying block size was performed. The research coordinator opened the next sequentially (in number) sealed randomization envelope after the patient had provided informed consent; the surgeon had ascertained that the patient met the eligibility criteria and that the implant types were all feasible for the patient. Both the patient and the staff on the ward were blinded to the implant allocation used during rehabilitation. The physiotherapist who conducted the follow-up visits (at 2–3 months, 1 year, and 2 years, i.e., the outcome assessor) was also blinded to the allocation. The patients did not receive any information on the specific implant design used in their operation until all patients had completed the 2-year follow-up visit.

### Statistics

Descriptive statistics, including mean and standard deviation (SD) for continuous variables, was used. The primary outcome analysis was an assessment of the change score in respective PROMs. Treatment effects were estimated with a linear mixed model. We performed a full data set analysis, which included outcomes at 3 months, 12 months, and 24 months as independent values. The repeated measures analysis was performed for the OKS, the FJS, the 15D, and the UCLA. The patient is a random factor, and group–time interaction and any covariates are fixed factors. Respective baseline values and age and sex were used as covariates. Marginal estimates for group-time interactions at each time point with associated P values and 95% confidence intervals (CI) were used as the primary results. The Satterthwaite method was used for degrees of freedom approximation. A chi-square test was used to compare binary and ordinal outcomes at each time point.

Initial analysis was done blindly. After blinded interpretation of the numerical results, we re-ran the analysis and used the intervention (Persona) group as a reference value for marginal group difference estimates. Rstudio v4.0 (R Core Team; R Foundation for Statistical Computing, Vienna, Austria) was used for the analysis.

### Ethics, registration, data sharing, use of AI, funding, and disclosures

The study protocol was reviewed and approved by the Regional Ethics Committee of Pirkanmaa Hospital District (R15053) and registered in the ClinicalTrials Registry (NCT03339557). Informed written consent was obtained from all patients. The datasets analyzed are available after ethical approval and from the corresponding author on reasonable request. We did not use AI at any stage. The study was financially supported by the Competitive State Research Financing of the Expert Responsibility area of Tampere University Hospital. Complete disclosure of interest forms according to ICMJE are available on the article page, doi: 10.2340/17453674.2024.42708

## Results

**Figure 1:**
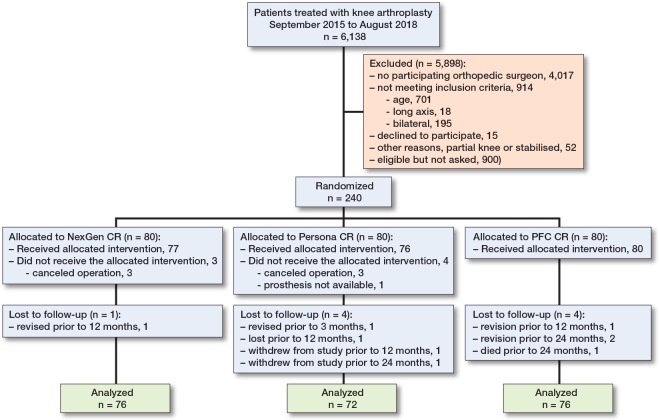
Flowchart of patient recruitment.

Between September 2015 and August 2018, 240 patients were randomized to undergo TKA with a novel TKA implant design (Persona CR, 80 patients) or with 1 of 2 conventional TKA designs (NexGen CR, 80 patients, PFC CR, 80 patients) (Figure 1). Most of the patients were female (67.5%), with a mean (SD) age of 61.9 (5.6) years. After many visits were delayed from the beginning of 2020 due to the COVID-19 pandemic, the final 24-month assessments were completed in January 2021. Of the 240 patients who underwent randomization, 225 participated in the final follow-up visit and were included in the final analyses. The baseline characteristics were similar in the 3 treatment groups (Table 1). Femoro-tibial size combinations are presented below as Supplementary Tables. In our study, 22% (28) of the combinations from the possible 126 femoro-tibial size combinations were used in the intervention group, 13% in the NexGen group, and 76% in the PFC group.

**Table 1:** Baseline characteristics of the patients by study group. Values are mean (standard deviation) unless otherwise specified

Factor	Persona	NexGen	PFC
Age	62 (5.1)	62 (5.6)	62 (5.7)
range	51–70	49–71	49–70
Sex, n (%)			
Female	54 (68)	53 (66)	55 (69)
Male	26 (32)	27 (34)	25 (31)
ASA class, n (%)			
1	16 (20)	13 (16)	17 (21)
2	50 (63)	53 (66)	51 (64)
3	14 (18)	14 (18)	12 (15)
Side, n (%)			
Left	40 (50)	40 (50)	40 (50)
Right	40 (40)	40 (50)	40 (50)
BMI	33 (5.9)	31 (4.9)	31 (5.8)
range	18–49	20–48	21–46
Mechanical axis	2.7 (6.9)	3.6 (5.6)	3.5 (5.4)
range	–12 to 15	–12 to 15	–11 to 12
Extension deficit	2.5 (4.0)	2.3 (4.3)	4.1 (4.6)
range	–10 to 15	–15 to 10	–5 to 15
Flexion	107 (28)	111 (22)	113 (18)
range	60–140	70–140	85–140
OKS	23 (7.9)	23 (5.8)	22 (6.2)
median (range)	23 (8–45)	24 (8–36)	22 (7–38)
FJS	16 (15)	12 (11)	13 (11)
median (range)	14 (0–90)	11 (0–50)	11 (0–63)
BPI	59 (24)	55 (22)	63 (20)
median (range)	58 (0–106)	55 (0–104)	67 (16–99)
PCS	15 (12)	14 (11)	15 (10)
median (range)	12 (0–49)	14 (0–49)	13 (0–39)
UCLA	5 (1.3)	5 (1.4)	5 (1.3)
median (range)	5 (0–9)	5 (0–9)	5 (0–8)
15D – mean (SD)	0.84 (0.07)	0.82 (0.11)	0.84 (0.08)
median (range)	0.85 (0.7–1.0)	0.85 (0.1–0.9)	0.85 (0.6–1.0)

### Primary outcome

**Figure 2:**
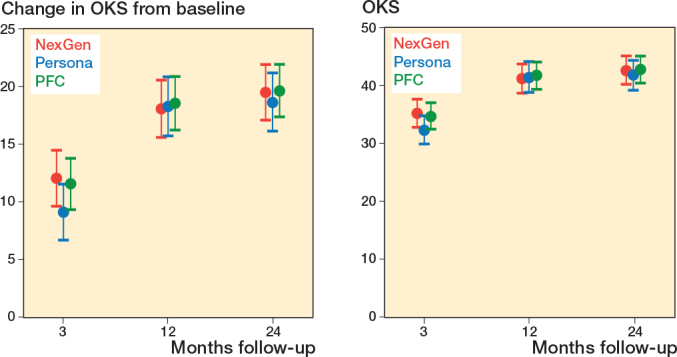
Change in OKS from baseline (primary outcome) and absolute OKS (secondary outcome) in the NexGen, the Persona, and the PFC groups. Values are marginal means with 95% CIs from the mixed model.

From baseline to 2 years, the OKS improved clinically significantly in all 3 treatment groups (Figure 2 and Table 2). The adjusted difference in change between PFC CR and Persona CR was 1.0 points (CI –1.7 to 3.6), favoring PFC CR. Similarly, the difference in change between NexGen CR and Persona CR was 0.9 points (CI –1.9 to 3.6). At the time of the final follow-up evaluation, the OKS was similar between the groups, as CI excluded between-group differences larger than 4 points (Table 3, see Appendix).

**Table 2:** Primary outcome Oxford Knee Score changes compared with baseline values at 3, 12, and 24 months after surgery, between-group difference results are adjusted for baseline values and age. Values are mean (standard deviation) and differences are mean (95% confidence interval)

Follow-up	Persona	PFC	NexGen	Between-group difference	
				PFC–Persona	NexGen–Persona
3 months	9.6 (9.7)	12 (8.5)	12 (7.2)	2.4 (–0.2 to 5.0)	2.9 (0.2 to 5.6)
12 months	19 (7.9)	19 (6.6)	18 (7.6)	0.3 (–2.5 to 3.0)	–0.2 (–3.0 to 2.6)
24 months	20 (6.8)	20 (6.8)	19 (7.7)	1.0 (–1.7 to 3.6)	0.9 (–1.9 to 3.6)

**Table 3:** OKS at 3, 12, and 24 months after surgery, between-group difference results are adjusted for baseline values, age, and sex. Values are mean (standard deviation) and differences are mean (95% confidence interval)

Follow-up	Persona	PFC	NexGen	Between-group difference	
				PFC–Persona	NexGen–Persona
3 months	33 (8.6)	35 (6.5)	34 (7.0)	2.4 (–0.2 to 5.0)	2.9 (0.2 to 5.6)
12 months	42 (6.2)	41 (6.9)	41 (5.8)	0.3 (–2.5 to 3.0)	-0.2 (–3.0 to 2.6)
24 months	42 (7.3)	43 (6.1)	43 (4.9)	1.0 (–1.7 to 3.6)	0.8 (–1.9 to 3.6)

### Secondary outcomes

**Figure 3:**
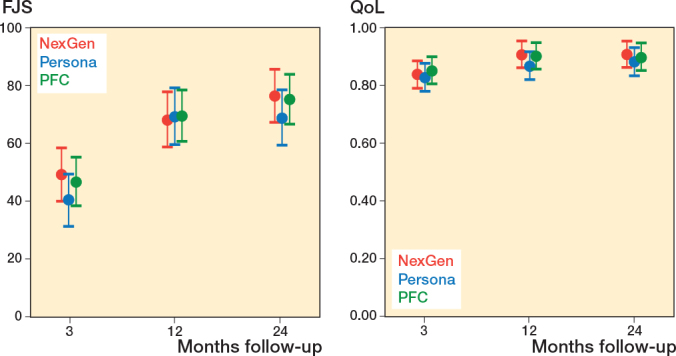
Secondary outcome (FJS and QoL) in the NexGen, the Persona, and the PFC groups. Values are marginal means with 95% CIs from the mixed model.

At the final follow-up, the change in FJS was lower in the Persona group compared with the NexGen and PFC groups (Figure 3, Table 4 and 5, see Appendix). This estimate was, however, imprecise and both zero difference and clinically relevant difference could not be excluded based on the CIs. Similarly, there was no difference in 15D and UCLA between the groups, which supports our null hypothesis. 4 patients assessed the outcome of the operation as poor and 15 patients rated the outcome as fair (Table 4).

**Table 4:** Changes in secondary outcomes comparing to baseline values at 3, 12, and 24 months after surgery, between-group difference results are adjusted for baseline values and age. Values are mean (standard deviation) and differences are mean (95% confidence interval)

Outcome Follow-up		Persona	PFC	NexGen	Between-group difference	
					PFC–Persona	NexGen–Persona
FJS	3 months	25 (24)	35 (24)	32 (26)	6.6 (–3.3 to 16)	9.3 (–0.8 to 19)
	12 months	54 (25)	54 (26)	55 (23)	0.5 (–9.7 to 11)	–0.5 (–11 to 10)
	24 months	53 (27)	62 (25)	61 (23)	6.5 (–3.4 to 16)	8.0 (–2.2 to 18)
15D	3 months	0.03 (0.07)	0.05 (0.15)	0.01 (0.14)	–0.01 (–0.05 to 0.03)	0.01 (–0.03 to 0.05)
	12 months	0.04 (0.12)	0.08 (0.12)	0.06 (0.07)	0.03 (–0.01 to 0.07)	0.03 (–0.01 to 0.07)
	24 months	0.06 (0.07)	0.08 (0.12)	0.06 (0.1)	0.01 (–0.03 to 0.05)	0.01 (–0.03 to 0.05)
UCLA	3 months	0.5 (1.5)	0.3 (1.5)	0.5 (1.5)	0.03 (–0.52 to 0.59)	0.18 (–0.41 to 0.77)
	12 months	1.5 (1.6)	0.9 (1.7)	1.4 (1.7)	–0.02 (–0.62 to 0.59)	–0.19 (–0.81 to 0.44)
	24 months	1.5 (1.5)	1.3 (1.8)	1.6 (1.8)	0.15 (–0.43 to 0.74)	0.19 (–0.41 to 0.8)
Anchor question, n (%)**^a^**						
	Excellent	35 (60)	32 (48)	39 (57)		
	Good	18 (31)	26 (39)	25 (36)		
	Fair	4 (7)	6 (9)	5 (7)		
	Poor	1 (2)	3 (4)	0		

a “How would you rate the outcome of the operation?”

**Table 5:** Secondary outcomes at 3, 12, and 24 months after surgery. Values are mean (standard deviation) and differences are mean (95% confidence interval)

Outcome Follow-up		Persona	PFC	NexGen	Between-group difference	
					PFC–Persona	NexGen–Persona
FJS	3 months	40 (25)	47 (24)	45 (25)	6.3 (–3.3 to 16)	8.7 (–1.3 to 19)
	12 months	70 (26)	67 (27)	68 (24)	0.2 (–10. to 10)	–1.1 (–12 to 9.4)
	24 months	68 (28)	76 (26)	74 (23)	–1.2 (–11 to –8.5)	7.4 (–2.8 to 18)
15D	3 months	0.83 (0.19)	0.83 (0.21)	0.86 (0.15)	0.02 (–0.03 to –0.08)	0.01 (–0.04 to 0.06)
	12 months	0.87 (0.13)	0.90 (0.08)	0.91 (0.08)	0.02 (–0.03 to 0.07)	0.04 (–0.01 to 0.09)
	24 months	0.89 (0.09)	0.90 (0.08)	0.90 (0.11)	0.02 (–0.04 to 0.07	0.03 (–0.03 to 0.08)
UCLA	3 months	5.2 (1.4)	5.5 (1.2)	5.2 (1.2)	0.03 (–0.52 to 0.59)	0.18 (–0.77 to 0.41)
	12 months	6.2 (1.6)	6.3 (1.6)	6.2 (1.5)	–0.02 (–0.62 to 0.59)	–0.19 (–0.81 to 0.44)
	24 months	5.8 (2.2)	6.2 (2.3)	6.3 (1.5)	0.15 (–0.43 to 0.74)	0.19 (–0.41 to 0.80

### Adverse events

The proportion of adverse events was 8/75 in the Persona group, 11/71 in the NexGen group, and 9/78 in the PFC group (Table 6). When the Nexgen group was compared with the Persona group, the risk difference was 4.9% (CI –6.1 to 15.8). For PFC the risk difference was 0.1% (CI –9.1 to 10.8). Revisions were performed once in the Persona group and the NexGen group and 3 times in the PFC group (Table 7, see Appendix).

**Table 6:** Complications, adverse events, and reoperations

Adverse event	Persona (n = 8/76)	NexGen (n = 11/72)	PFC (n = 9/76)
Hematoma, bleeding		1	
Superficial wound infection (per os antibiotics)	2	2	
Postdural puncture headache	1	1	
Pulmonary embolus		1	
Death		1	1
Atrial fibrillation			1
Intraoperative fracture (conservatively treated)	1		
Manipulation under anesthesia	2	2	4
Superficial wound problem (no need for arthrotomy)	1	1	
Quadriceps tendon rupture		1	
Revision surgeries	1	1	3

**Table 7:** Reoperations

Item	Persona (n = 1)	NexGen (n = 1)	PFC (n = 3)
Prosthetic joint infection	61 year-old female, at 5 months, G streptococci group G, debridement, and implant retention DAIR followed by i.v. + per os antibiotics for 6 weeks		62 year-old male, at 7 months, possible low-virulent bacteria, 1-stage revision followed by 6 weeks of antibiotics
Secondary patellar resurfacing		69 year-old female, at 10 months, anterior knee pain, patellar resurfacing	51 year-old female, at 22 months, anterior knee pain, patellar resurfacing and exchange of polyethylene insert
Instability			66 year-old female, at 19 months, hyperextension and instability, revision to a CCK implant combined with patellar resurfacing

## Discussion

The aim of our RCT was to investigate the functional outcomes of a novel TKA implant design compared with 2 conventional TKA designs. Primary outcome was the change in OKS at 2 years. Secondary outcomes were FJS, 15D quality of life questionnaire, UCLA activity score, and complications.

The most important finding of this study was that patients treated with the novel TKA design achieved similar but not improved functional outcomes or quality of life at 24 months compared with controls. There was also no clinically relevant difference in the incidence of complications and reoperations between the implant designs.

This is the first RCT to study the clinical significance of a novel TKA using asymmetrical tibial components and finer sizing for the tibial liner. We were unable to observe any clinically relevant differences between the new and the 2 standard TKA designs. We defined MCID as a 5-point difference in the OKS at 24 months postoperatively and thus as a clinically significant difference between the studied groups, which were close to the predefined MCID. Patients showed neither floor nor ceiling effect. We did not observe any difference in the risk of revision; however, the study was not powered for this estimate but for PROMs. Keiller et al. (2023) found no differences in clinical outcomes but minor differences in migration pattern of the tibial component at 2 years between the primary Attune knee and PFC Sigma [21]. The Attune tibial component used in their randomized trial was replaced with a new version in 2017. The survival of 3 knee implants in our study has been shown to be excellent in long-term follow-up, as reported by many national joint registries [22-24].

The findings of our study support the fact that the proposed benefits with a larger range of component sizes and combinations in novel implants by implant manufacturers do not exist.

The outcomes of TKA are believed to result from surgeon-, patient- and implant-related factors [25]. Some benefits may be achieved with pain neuroscience education (PNE) in postoperative pain management after TKA [26]. Indeed, between 10% and 20% of patients who undergo TKA are to some extent dissatisfied with the outcome [27] and the same numbert of patient rated the outcome of the operation as fair or poor in our study. The reason for this dissatisfaction is poorly understood, but the proposed etiological factors include unmet patient expectations, psychosocial reasons, component malorientation, soft-tissue imbalance, non-anatomic implant shape, and poor implant fit. To address these factors, new, personalized and more anatomically accurate implants have been developed. These novel implants are designed to have improved implant shape and fit, thus aiming to improve function and satisfaction due to better joint perception.

### Limitations

First, we were not able to report the exact number of patients assessed for eligibility or the reasons for exclusion from the outpatient clinic. Second, the primary outcomes of this study depended on the PROMs and using more objective assessment methods may produce slightly different results and a better understanding of the true activity levels of TKA patients. Third, all surgeons were more familiar with the conventional implant systems. All orthopedic surgeons who participated in the study were high-volume arthroplasty surgeons. They had performed TKAs with control group implants for many years and performed at least 15 TKAs with Persona implants before the study population. Participating orthopedic surgeons had an annual volume of 200–300 arthroplasties. Fourth, in addition to CR inserts, ultracongruent and curved plus inserts were also used, leaving a possible larger variation compared with the use of only CR inserts in all implants. Finally, although 2 years is too short a follow-up time to evaluate the survival of a certain TKA implant, it is long enough for the comparison of PROMs. The strengths of this study were the original double-blinded RCT design, the range and consistency of the outcome measures employed, and that the study was sufficiently powered to detect a difference in the OKS outcome score.

### Conclusion

We found no differences in change in functional outcomes measured regarding primary OKS, and secondary 15D, or the FJS between 2 conventional TKR implant designs and a novel implant design at 2-year follow-up.

*In perspective,* conventional designs with a proven track record of survivorship and function can offer the same patient-reported and functional outcomes as novel designs. There is a need for better evaluation of new implants before introduction to the market.

## Supplementary Material



## Supplementary data

Supplementary Table is available as supplementary data on the article page, doi: 10.2340/17453674.2024.42708

## Notes

Design of the study: JK, AE, MN, VMM, TM. Drafting the work and analysis: TI, AR. Final approval of the version to be published: TI, AR, JK, AE, MN, VMM, TM. Agreement to be accountable for all aspects of the work in ensuring that questions related to the accuracy or integrity of any part of the work are appropriately investigated and resolved: TI, AR, JK, AE, MN, VMM, TM

The authors would like to thank Peter Heath for his contribution.

Handling co-editors: Marianne Westberg and Robin Christensen

*Acta* thanks Mona Badawy and Martin Lindberg-Larsen for help with peer review of this manuscript.
