# Supplementary material for: Assessment of improvement in functional outcomes between a novel knee replacement design and conventional designs in 240 patients: a randomized controlled trial
Source: Acta Orthop. 2025 Jan 24;96:127–34. doi: 10.2340/17453674.2024.42708 (PMC11760186; doi:10.2340/17453674.2024.42708)
Supplement: Supplementary file 1 [file ActaO-96-42708-s1.pdf]

Supplemental Table 1. Rows representing the size of the tibia component and the columns are for the size of the femur component (n = narrow), colored cells represent femoral and tibial compatibility

Femoro-tibial size combinations in the intervention group and femoral/CR bearing and tibial compatibility

	1n	1	2n	2	3n	3	4n	4	5n	5	6n	6	7n	7	8n	8	9n	9	10n	10	11n	11	12n	12
A																								
B																								
C							10 mm CR				10 mm CR<br>10 mm UC	2 x10 mm CR												
D										2 x 10 mm UC<br>12 mm CR	10 mm CR	3 x 10 mm UC	13 mm UC	10 mm CR<br>12 mm UC	2 x 10 mm CR<br>2 x 11 mm CR		10 mm CR							
E												10 mm UC<br>11 mm UC<br>13 mm CR	PS	2 x 10 mm CR<br>10 mm UC<br>11 mm CR<br>12 mm UC<br>2 x 13 mm UC<br>13 mm CR<br>14 mm UC	11 mm CR<br>11 mm UC	2 x 10 mm CR<br>11 mm CR<br>11 mm UC<br>12 mm CR<br>16 mm UC	10 mm UC<br>12mm CR							
F												10 mm CR<br>12 mm CR		2 x 10 mm UC<br>11 mm UC<br>16 mm UC	11 mm UC<br>2 x 12 mm CR	10 mm CR<br>2 x 10 mm UC<br>11 mm CR<br>11 mm UC				10 mm CR<br>12 mm CR		10 mm UC<br>11 mm CR		
G																		10 mm CR<br>11 mm CR<br>11 mm UC<br>12 mm CR		10 mm CR		10 mm CR<br>2x 11 mm CR		
H																		12 mm CR				2 x 10 mm CR<br>14 mm CR		10 mm CR
J																								

Femoro-tibial size combinations in the control group (NexGen)

	A	B	C	C GSF	C-	C- GSF	D	D GSF	D-	D- GSF	E	E GSF	E-	E- GSF	E LPS	F	F GSF	F-	F- GSF	F LPS	G	G GSF	G-	G- GSF	H
1																									
2							2 x 12 mm	10 mm		17 mm		12 mm													
3			10 mm<br>Allpoly				4 x 10 mm<br>Allpoly<br>3 x 12 mm	10 mm<br>Allpoly			3 x 10 mm<br>Allpoly<br>4 x 12 mm	5 x 10 mm<br>Allpoly	14 mm			10 mm<br>Allpoly	10 mm<br>Allpoly								
4											2 x 10 mm<br>Allpoly<br>2 x 12 mm<br>14 mm	10 mm<br>Allpoly				10 mm<br>Allpoly	10 mm<br>Allpoly		12 mm						
5											2 x 10 mm<br>Allpoly<br>3 x 12 mm<br>14 mm				12 mm	5 x 10 mm<br>Allpoly<br>2 x12 mm	2 x 10 mm<br>Allpoly	12 mm	10 mm<br>Allpoly	12 mm	10 mm<br>Allpoly<br>12 mm				
6											10 mm<br>Allpoly					10 mm<br>Allpoly<br>2 x 12 mm<br>14 mm		10 mm<br>Allpoly			3 x 10 mm<br>Allpoly<br>12 mm				
7																2 x 10 mm<br>Allpoly<br>2 x 12 mm<br>Allpoly<br>14 mm					2 x 10 mm<br>Allpoly<br>12 mm<br>Allpoly				
8																									12 mm
9																									12 mm
10																									

Femoro-tibial size combinations in the control group (PFC)

	2	2.5	3	4 N	4	5	6
2	8 mm plus<br>12.5 mm	8 mm plus<br>10 mm					
2.5		8 mm Allpoly<br>8 mm plus<br>10 mm<br>3 x 12.5 mm plus	8 mm<br>2 x 8 mm plus<br>2 x 10 mm<br>3 x 10 mm plus<br>4 x 12.5 mm<br>2 x 12.5 mm plus				
3		10 mm plus	8 mm<br>2 x 10 mm<br>5 x 10 mm plus<br>15 mm	8 mm plus<br>5 x 10 mm<br>4 x 10 mm plus<br>3 x 12.5 mm	2 x 10 mm<br>3 x10 mm plus<br>12.5 mm		
4			8mm Allpoly		4 x 10 mm Allpoly<br>5 x 10 mm plus Allpoly	8 mm Allpoly<br>5 x 10 mm Allpoly<br>2 x 12.5 mm Allpoly<br>15 mm Allpoly	
5						10 mm<br>2 x 10 mm plus<br>2 x 12.5 mm	3 x 10 mm plus
